# Exergames and Immersive Virtual Reality as a Novel Therapy Approach in Multiple Sclerosis: Randomised Feasibility Study

**DOI:** 10.3390/jcm13195845

**Published:** 2024-09-30

**Authors:** Gustavo Rodríguez-Fuentes, Elena Ferreiro-Gómez, Pablo Campo-Prieto, José Mª Cancela-Carral

**Affiliations:** 1 Departamento de Bioloxía Funcional e Ciencias da Saúde, Facultade de Fisioterapia, Universidade de Vigo, E-36005 Pontevedra, Spain; gfuentes@uvigo.gal; 2 HealthyFit Research Group, Galicia Sur Health Research Institute (IIS Galicia Sur), SERGAS-UVIGO, E-36312 Pontevedra, Spain; chemacc@uvigo.gal; 3 Fisionova, Physiotherapy Center, E-36002 Pontevedra, Spain; elenafego22@gmail.com; 4 Departamento de Didácticas Especiais, Facultade de Ciencias da Educación e do Deporte, Universidade de Vigo, E-36005 Pontevedra, Spain

**Keywords:** rehabilitation, exercise, virtual reality exposure therapy, Multiple Sclerosis, wearable technology, physiotherapy

## Abstract

**Background:** Multiple sclerosis is a chronic, inflammatory, neurodegenerative autoimmune disease caused by myelin loss in the central nervous system, which leads to motor and non-motor problems. The main objective of this study was to explore whether an immersive virtual reality (IVR) exercise programme would be feasible as a form of physical therapy for people with MS (pwMS). **Methods**: 18 participants (13 women; 45.06 years) were assigned to an experimental group (EG, n = 8) and a control group (CG, *n* = 10). The EG took part in a twice-weekly IVR exergame physical therapy programme—ExeRVIEM programme. A randomised, single-blind clinical trial was conducted and was registered in clinicaltrials (NCT05870254). **Results**: The intervention was feasible and safe. The participants completed the programme with no adverse effects (no symptoms on the Simulator Sickness Questionnaire), high usability (System Usability Scale 90.31%), and outstandingly positive post-game experiences (Game Experience Questionnaire 3.10/4). In addition, the GE significantly improved several of their functional capacities: increased lower limb strength (Five Times Sit-to-Stand Test *p* = 0.042), improved functional mobility, and reduced fall risk (Timed Up and Go Test-simple *p* = 0.009; Timed Up and Go Test-cognitive *p* = 0.003). There were no statistically significant differences between the groups. **Conclusions**: The findings support that the use of exergames and IVR as physical therapy in pwMS is feasible and safe. Furthermore, there is the suggestion of possible benefits to participants’ functional abilities, all of which position IVR as a promising tool for the rehabilitation of this neurodegenerative pathology affecting young adults.

## 1. Introduction

In 2020, the prevalence of multiple sclerosis (MS) was estimated to be 35.9 per 100,000 people, making it one of the most predominant central nervous system diseases [1] and the most common non-traumatic disease in young adults globally [2].

MS is caused by myelin loss in the central nervous system, resulting in an inflammatory, chronic, neurodegenerative autoimmune disease [3]. It usually begins in the third decade of life [4] and can progress in the form of outbreaks or episodes of neurological dysfunction [5] with an unpredictable course [6], leading to the appearance of various motor and non-motor symptoms. The most common are [3] sensory disturbances (numbness, tingling), difficulties in walking (due to fatigue, weakness, spasticity, lack of balance, lack of coordination, and tremors) that increase the risk of falling, sight problems, constipation, bladder dysfunction, learning difficulties, depression, dizziness, vertigo, and sexual dysfunction. Additionally, according to the study by Walton et al. [1] on the update of MS prevalence data carried out in 2020, there was a 30% increase in the prevalence of MS following the same methodology that had been used in 2013 [5], reflecting the significance both in terms of public health and of healthcare costs that this disease may involve going forward.

The lack of adequate treatment for this prevalent health problem means that other non-pharmacological therapeutic strategies must be added to the arsenal of those already used in order to reduce the many problems caused by the symptomatology described above. One of the strategies that shows evidence of numerous benefits is therapeutic exercise [7]. In patients with MS, therapeutic exercise leads to benefits in gait [8], balance [9], cardiorespiratory function [10], fatigue [11], musculoskeletal function [10], blood levels of brain-derived neurotrophic factor [12], cognition [13], neurogenic bladder symptoms [14], symptoms of depression [15] and quality of life [16]. However, these benefits do not always occur, as reflected in the review by Malone et al. [10], where some of the studies considered did not demonstrate significant benefits related to fatigue or symptoms of depression, and others where there were still knowledge gaps about the appropriate “dose” of therapeutic exercise needed when seeking to improve balance [17].

Additionally, therapy based on repetitive and monotonous physical exercise can sometimes be unmotivating for patients. Furthermore, it has been considered a controversial therapy, as it was believed that it could exacerbate the symptoms of MS [18]. These aspects may have influenced the degree of compliance and motivation towards exercise therapies [19]. On the other hand, Immersive Virtual Reality (IVR) may, therefore, be able to offer exercise-based programs (using exergames) that are more motivating and entertaining while at the same time being appropriate to the therapeutic objectives of these patients. This tool has already been successfully tested in other groups (post-stroke, Parkinson’s, etc.) with promising results [20,21].

In recent studies, virtual reality (VR) appears to have had beneficial effects in the treatment and rehabilitation of people with MS (pwMS). A systematic review with meta-analyses showed that treatment carried out with VR appears to be effective in improving balance and gait quality in pwMS, providing a valid, motivating, and apparently more effective alternative to conventional therapies [22]. A randomised clinical trial found that VR, in addition to improving the functional mobility and balance of these patients, also reduced fatigue—one of the main and most debilitating symptoms of MS—suggesting its potential as a new therapeutic tool in the rehabilitation of pwMS [23]. These findings highlight the importance of further research into the application of VR for the optimisation of clinical outcomes in the MS population, as well as for improving the quality of data available for these devices.

This proposal, based on IVR exergaming by pwMS, is a strategy to improve the functionality of patients with this pathology, where patients will carry out an exercise programme using a virtual reality headset. The rationale for using the IVR is that, in addition to improving functionality, it may motivate patients and encourage their adherence to the treatment programme, as other studies have previously shown [24]. Furthermore, the hope is that not only will the programme lead to health benefits for MS patients but also that the selected, controlled and supervised use of IVR, along with our previous experience with this type of application, will minimise the possibility of adverse symptomatology that exposure to IVR may generate.

Therefore, the main objective of this pilot study is to test the feasibility and safety of carrying out a therapeutic exercise programme based on IVR in pwMS, as well as to investigate—as a secondary objective—what effects, and to what extent, this intervention generates at a physical and functional level, in this population.

## 2. Materials and Methods

### 2.1. Study Design

A randomised, single-blind clinical trial, in which the evaluator did not know to which group each person evaluated belonged. The participants were randomly with IBM-SPSS v25.1 software and divided into a control group (CG) and an experimental group (EG). The study has been registered in clinicaltrials.gov (NCT05870254 accessed on 25 June 2024) and its protocol has been previously published [25].

### 2.2. Participants

**Figure 1:**
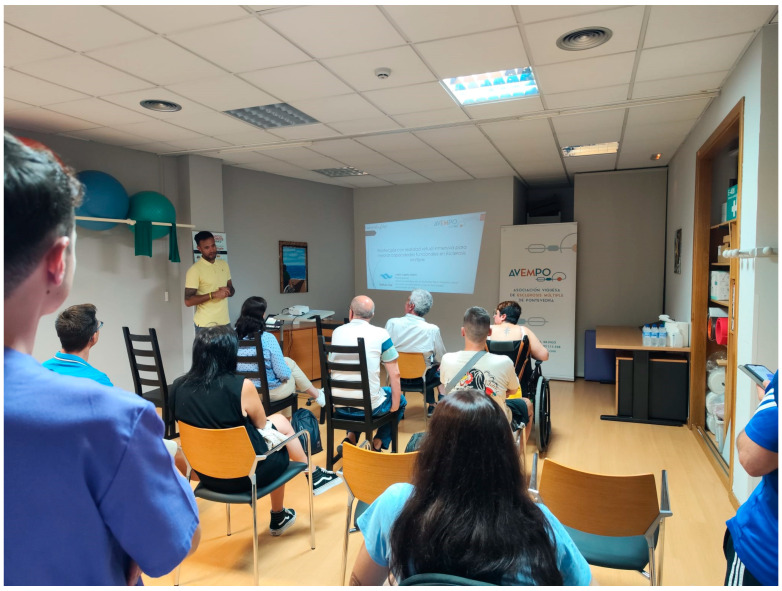
Information session for people with Multiple Sclerosis.

The participants in this study are all members of the AVEMPO Association, based in Vigo, Spain. All the members of the Association were informed about and invited to participate in the study at an information session held by the researchers. To this end, the staff members provided an informed consent form so that both the participants and their families could read it at home. They were asked to return it signed within 10 days. No incentives were offered. Those who were interested in participating, met the selection criteria and signed the consent were included in the trial (see Figure 1).

The inclusion criteria were as follows: an MS diagnosis, being members of the Association, being aged between 18 and 65 years, and being able to stand safely and follow the intervention protocol and scheduled assessments. The exclusion criteria were as follows: having a medical report that advised against physical activity and exercise, being subject to an uncontrolled outbreak of the disease, or experiencing dizziness, vertigo or severe visual impairment.

**Figure 2:**
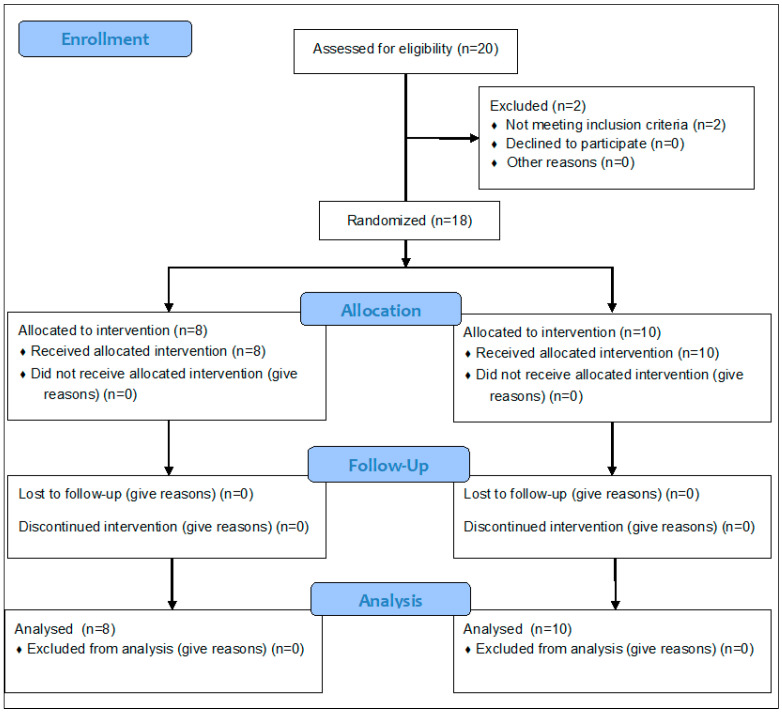
Study design: CONSORT 2010 Flow Diagram.

The final sample consisted of 18 participants, who were random with IBM-SPSS v25.1 software and divided into a CG, with 10 participants, and an EG, with 8. The CG continued with their usual therapies at the centre, while the EG, in addition to these, carried out the IVR programme. Figure 2 graphically depicts the trial design following the CONSORT 2010 guidelines [26].

The research was carried out after informed consent had been signed by all participants and was conducted in accordance with the ethical principles of the Declaration of Helsinki [27], as well as the provisions established on Personal Data Protection (Organic Law 3/2018) and on the Guarantee of Digital Rights (Organic Law 3/2018, of 25 May), and after having been approved by the University of Vigo Faculty of Physiotherapy Ethics Committee (code 205-2023).

### 2.3. IVR Device and Software

The Meta Quest III VR headset (Oculus VR) was used. This is a portable device consisting of a wireless headset and two controllers, which are simply connected to a Wi-Fi network. To increase user comfort, an Elite Strap was added to improve the ergonomics of the device. A laptop computer was also used so the therapist monitoring the sessions could simultaneously observe the same images being viewed by the participant in their headset. In this way, the patient’s movements could be safely guided.

For the IVR intervention, Les Mills software (1.9.0 version) was used (available in the library at www.meta.com on 25 June 2024), a boxing-based exergame that simulates being in a gym. In the user’s field of vision are two boxing gloves with a different colour for each hand, which they have to use to “punch” various “objects”. The participants have to react to the different stimuli presented to them, with different physical movements. These movements involve the upper limbs, lower limbs, trunk and head, thus generating a comprehensive workout.

**Figure 3:**
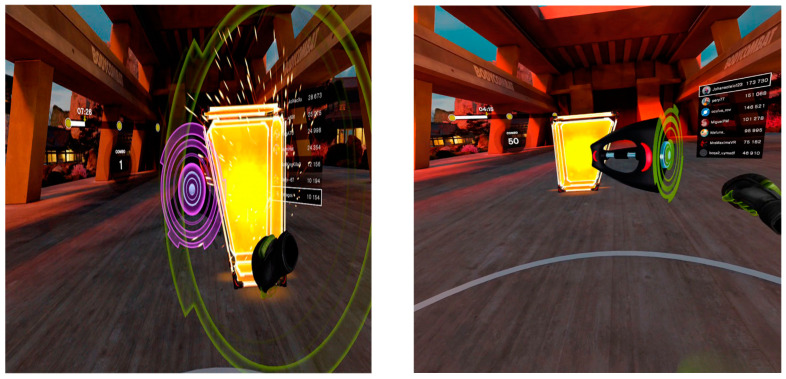
Screenshots of the exergame with examples of direct hit, crochet and dodge platforms in yellow.

There are two distinct kinds of movement that are encouraged by the different stimuli in the exergame. Firstly, punching, where the participants have to extend their upper limbs towards the illuminated punch balls that approach them and match the colours of their gloves with the colours of the punch balls. The punches are of different types: jabs (frontal), crochet (outside–inside) and hook (down and up). Secondly, there are dodges, where the participants have to avoid touching blocks that come towards them by moving their weight from side to side (side blocks) or up and down by doing squats (upper blocks) (see Figure 3).

The total score is the result of the number of successful hits and dodges of the blocks, the points of which increase as hit streaks are completed and if the targets are hit with greater force, speed or range of motion. This programme was selected because of its low difficulty and short duration, thus decreasing the chances of any adverse effects. Even so, patients were told to stop the intervention at any time if they experienced discomfort.

**Figure 4:**
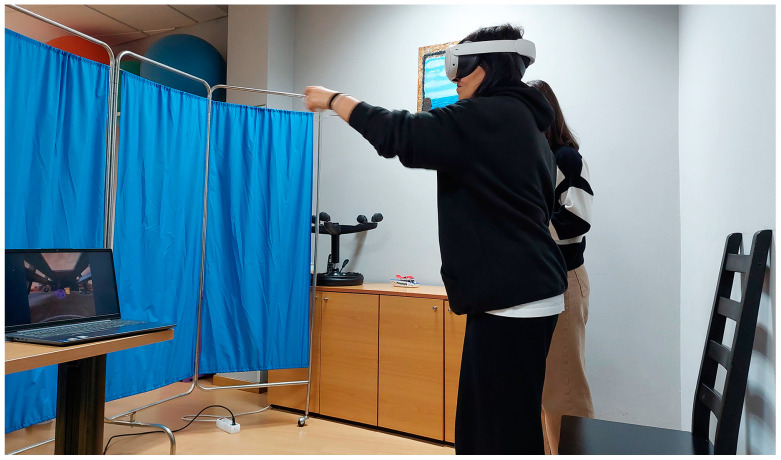
Example of a participant carrying out a session of the ExeRVIEM programme.

The sessions were individualised and supervised by a physiotherapist, ensuring the well-being and safety of the patients during the programme (see Figure 4).

### 2.4. Intervention Program

The experimental group carried out the ExeRVIEM program, which consisted of two IVR sessions a week over 8 weeks, at least 48 h apart, in addition to continuing with all their usual treatment as prescribed by the AVEMPO Association rehabilitation team. The IVR programme with the Les Mills software (1.9.0 version) lasted 5 min and 27 s and was added to the end of the patients’ normal physiotherapy sessions. After having completed the session, calm breathing cycles were performed in a seated position while the intervention data were collected. Thus, the total duration of the session was approximately 10 min.

Meanwhile, the CG continued with its usual therapeutic programme, as prescribed and administered by the Association, which contained occupational therapy and physiotherapy sessions.

### 2.5. Evaluation Tools

A baseline assessment was performed initially, followed by another one at the end of the intervention. In addition to the assessments, demographic and clinical data (gender, age, co-morbidities, years since diagnosis, MS subtype and pharmacological treatment) were collected.

In order to observe possible effects on the physical aspects of the intervention, all participants underwent the following tests and questionnaires:

- Tinetti Test to assess gait and balance, as well as to determine the level of early-stage fall risk [28];
- Five Times Sit-to-Stand Test (FTSST) to assess the functional mobility and strength of the lower extremities [29];
- Timed Up and Go Test to assess mobility, dynamic and static balance and fall risk [30,31]. This was carried out in its basic form (TUG-simple) and also by performing a double task (TUG-cognitive), in this case, performing the test whilst simultaneously doing mathematical operations;
- Fatigue Severity Scale (FSS) to assess the participants´ level of fatigue [32];
- Handgrip Strength Test to quantify maximum isometric hand and forearm strength [33,34]. A Jamar^®^ Smart digital hand dynamometer (J.A. Preston Corporation, Clifton, NJ, USA) was used;
- Reaction Time was measured using Rezzil software (1.9.0 version), which is accurate to a thousandth of a second when assessing the time it takes for a subject to react to the appearance of stimuli [35].

In addition, at the final evaluation, patients belonging to the EG completed the following questionnaires related to the intrinsic aspects of IVR exposure:

- Simulator Sickness Questionnaire (SSQ). This assesses the safety of the experience. It consists of 16 items grouped into 3 subscales and further divided by symptomatology: 1. Oculomotor symptoms; 2. Disorientation. 3. Nausea. Each item is evaluated on a four-point scale (0 = do not feel anything, 1 = a little, 2 = medium and 3 = a lot), and the total score is the sum of the scores of the three subscales [36,37,38];
- System Usability Scale (SUS). This scale quickly and easily assesses the usability of the device/protocol. It consists of ten questions on a Likert-type scale. Each question is scored from 1 to 5 according to the level of agreement or disagreement with each statement, with 5 meaning completely agree and 1: completely disagree. The algorithm which results from these answers creates a score out of a maximum of 100 points [39,40];
- The post-game module of the Game Experience Questionnaire (GEQ-post game). This questionnaire assesses each participant’s personal, subjective experience. It consists of three modules (main module, social module and post-game module). Here the post-game module was used, which assesses how players feel after they stop playing the game. This module is also a Likert-type scale consisting of 17 items where responses are graded according to the intensity of the feelings experienced (0: not at all, and 4: extremely). These items are, in turn, divided into 4 components (positive experiences, negative experiences, tiredness and return to reality), which are scored individually and whose average could result in a maximum score of 4 points [41]. In the absence of a validated version of the questionnaire in Spanish, and so the current study group would have no problems with this questionnaire, a version was used which had already been translated by the authors and been used in previous research [42,43];
- Ad hoc satisfaction questionnaire. This questionnaire had previously been developed to identify the strengths and weaknesses of similar interventions. [20,42].

Finally, after the end of each IVR session, the perceived effort expended during the intervention was recorded with the modified Borg scale [44]; the presence or absence of cybersickness was noted, as was the total score achieved, as well as the percentage of correct scores in the game.

### 2.6. Data Analysis

An analysis of the sociodemographic characteristics of the sample was carried out using parameters of central tendency (mean) and dispersion (standard deviation) across both groups. Due to the size of the sample, its normality was verified using the Shapiro–Wilks test (*p* > 0.05), which showed a normal distribution, so parametric tests were used in the statistical analysis. The descriptive analysis of the main variables of each of the groups was carried out using mean and standard deviation. Levene’s test was applied to verify the homogeneity of the groups (both Experimental and Control) and found that the groups were statistically homogeneous. To compare the results for each group at the beginning and the end of the study, a paired-sample Student’s *t*-test for related samples was used. Differences in intergroup pre-test–post-test were determined using 2 × 2 ANOVA (groups × moment) with Bonferroni’s correction. All analyses were performed with IBM-SPSS v25.1 software, and a *p*-value of <0.05 was considered statistically significant.

## 3. Results

The study was carried out on a population of 18 subjects, whose demographic data are summarised below. The sample consisted of 5 men and 13 women, with a mean age of 45.06 years. The time from diagnosis was varied, ranging from 1 to 28 years, while the type of MS was predominantly relapsing–remitting, with only two cases of secondary progressive and two cases of primary progressive. These subjects were randomly assigned to two homogenous groups, each having no significant differences from the other at the start of the study (except in age and TUG-simple) and, therefore, being comparable.

In view of the findings, it should be noted that all participants completed the total number of IVR sessions. There was, therefore, 100% safe adherence to the programme, as no adverse effects were recorded.

The pre-intervention physical assessment data collected for both the EG and the CG are shown in Table 1.

With regard to results relating to the primary objective set out in Table 2 (feasibility and safety of the device), an excellent usability rating was scored in the SUS (90.31/100), while in the SSQ, very low scores were obtained (1.37/48). In all cases, these data point to very mild symptomatology within the expected range of normality.

As for the GEQ-post-game, outstanding results were obtained in the positive experiences (3.10/4), with no negative experiences being recorded at all, and with only very low values recorded in relation to fatigue (0.44/4) and return to reality (0.17/4) after exposure to the IVR. All these data indicate that the IVR device and software is a safe and user-friendly tool, as well as being perceived positively by patients.

The responses to the ad hoc questionnaire further confirm the results obtained in the previous scales. 100% of the subjects rated the experience positively, recommending the experience and explaining that they would repeat it as they had noticed improvements in their abilities and had fun at the same time.

Comparing the data from the first and the last session, a clear improvement can be seen in general aspects (Table 3). There is a higher score, a higher success rate and a lower perception of effort. This indicates that the performance of the proposed IVR task has improved while the perceived effort has decreased.

As for the results of the physical assessment (Table 4 and Table 5), and in response to the secondary objective, the Tinetti test did not demonstrate a significant improvement in either group, with only slight increases of 1.91% in the EG and 0.79% in the CG respectively. In the TUG and TUG-cognitive results, however, there was a significant improvement in the results of both tests for the EG, with an increase of 13.56% and 19.25%, respectively, in each test. For the CG, conversely, the mean data slightly deteriorated, by 8.11% in the normal TUG and 10% in the cognitive TUG. When the data obtained in the FTSST is analysed, significant improvements in the EG times can be seen, which diminished by 11.65%, while in the CG, they remained stable. In relation to manual grip strength, both groups registered an improvement in their performance, with 15.31% for the EG and almost 19% for the CG. As for the FSS results, small improvements were seen in both groups, these being 6.49% in the EG and 3.64% in the CG, as well as in the reaction time values, which improved by 16.52% in the EG and 8.41% in the CG.

Despite these aforementioned results, it should be noted that no significant differences were found between EG and CG after the intervention.

## 4. Discussion

The main objective of this study was to assess the feasibility and safety of using IVR hardware along with the Les Mills boxing exergame in pwMS, as well as to investigate its usability and the subjects’ own experiences. Based on the findings, we can state that an 8-week protocol with IVR does seem feasible and safe in this scenario. This is further corroborated by the fact that no adverse symptoms occurred after the use of IVR by the EG subjects, who successfully completed 100% of the sessions. These results are supported by other studies [45,46], which have also found no severe adverse effects related to the implementation of VR programmes in patients with MS. Additionally, these data reinforce the validity of IVR interventions in the promotion of better adherence to exercise therapy in patients [47,48].

Usability was excellent according to the scores on the SUS scale, which measures the usability of the system. This fact is important since, in the past, some barriers have been described that limit access to technology for this population [49]. Our results were gratifying as the SUS scores were higher than 85%, giving a mean usability of 90.31%, which is similar to results recorded in other studies [45,50]. In the study by Winter et al. [45], subjects with MS who completed IVR sessions scored an average of 83.75% on the SUS scale. Additionally, in the research by Kamm et al. [50], patients who performed physical training with IVR sessions autonomously at home scored 94% on the SUS scale. These data show how simple the IVR equipment is for pwMS to use. And as mentioned above, they could not only facilitate access to e-health but also facilitate access to exercise therapies in safe environments such as homes or patient associations.

As for the GEQ-post game scores, the results are promising. Positive elements scored highly, while negative elements and those related to fatigue and return to reality scored low. Together with the participants´ high adherence to treatment, these results indicate that the IVR programme under study here is a suitable option for initiating therapeutic exercise in MS patients. These positive results are complemented by the ad hoc satisfaction questionnaire, where 62.5% of the participants rated the experience as good or very good, and the remaining 37.5% rated it as enjoyable. All the comments were positive. Furthermore, 100% of the subjects belonging to the experimental group would repeat an IVR experience and recommend it to others. Similar results have already been observed in other research with older people [43] and in people with Parkinson’s [20]. However, there is still not enough literature about IVR experiences in patients with MS, so this study adds more evidence to a growing field of recent research.

In terms of functional capacities, post-intervention benefits were observed in both groups, though the IVR experience cannot be held entirely responsible for these. It is important to remember that both groups continued with their respective therapies in the Association, so that the IVR intervention, although positive, did not achieve a statistically significant improvement compared to the CG. Maybe future studies with a greater workload requirement for patients or a longer intervention duration, now that we know that this approach was feasible and safe, could achieve the necessary level of effort to result in a significantly greater improvement compared to the group that did not do IVR. However, the positive results are greater in the EG, which could be explained by a learning-by-training phenomenon, by the improved physical condition of the participants and/or by the influence of the IVR: offering fun scenarios [20], gamified therapies [35] or reducing pain [24] or the perception of effort [51], among others.

Continuing with the secondary objective of this pilot study, regarding physical and functional improvements, other studies have had success with different patient groups by doing exercise with IVR [35,42,52]. Campo Prieto et al. [35] effectively focused on reducing reaction time in Parkinson’s patients, thus contributing to a lower risk of falls. Similarly, functional improvements in gait, balance and handgrip strength parameters have been achieved in institutionalised older adults [42]. IVR has also been shown to be effective for stroke patients, providing greater benefits in the recovery of upper limb motor function and autonomy in the activities of daily living than conventional therapy alone [52]. This demonstrates the possibility and importance of achieving these physical and functional improvements in people with MS, as has been accomplished in similar groups and populations.

It is important to note the studies which indicate that patients with MS experience a decrease in muscle strength of up to 70% when compared to healthy subjects [53]. Additionally, they suffer from balance impairment and a delay in the different motor responses, which reduces their ability to move and the quality of walking, significantly increasing their risk of falls [54,55]. These difficulties are aggravated when a cognitive task is added to the motor task [56]. Therefore, the significant improvements found in the EG of this pilot study in both the FTSST and the normal and cognitive TUG tests—which assess lower limb strength, functional mobility and the risk of falls, respectively—are of particular importance for participants in this group, because their quality of life and health status could be significantly improved as a result. Furthermore, although there were no statistically significant intergroup differences in any variable, it cannot be overlooked that, in the TUG, the IVR group registered an improvement of more than 1.32 s, which is the minimum detectable change that can be considered a true change [31], something not seen in the control group. On the contrary, the TUG values in both modalities had deteriorated in the CG´s post-intervention measurement.

In terms of levels of perceived exertion, as measured on the modified Borg scale, these were 2.37/10 on average at the end of the last session. This scale has been shown to be a valid and reliable tool for research and medical diagnosis [57]. Therefore, it could be inferred that this sample would be ready to face a more demanding programme if, at first, their safety in performing the tasks was prioritised. Furthermore, the data are in line with other studies which show that IVR exercise programmes tend to decrease the perception of effort without a resulting reduction of energetic output [58,59]. This could be very beneficial for these types of patients and also suggests that it may be safe to increase the workload carried out with IVR (either by increasing the number of sessions and/or their duration) and, thus, the potential benefits of IVR-based therapeutic exercise.

### Limitations

Some limitations of this study should be noted. Firstly, the results obtained cannot be generalised to the population, as the sample size of the pilot study is not representative. Since the primary objective of the research was successfully achieved and the IVR proved to be a safe and feasible tool for this type of population, further studies should be conducted with a larger sample size in order to generalise the data obtained. Additionally, the duration of the research was limited, requiring future research studies with longer interventions, in which the sample is also followed up to assess whether the results obtained are maintained over time and, if so, for how long. Finally, although in this case, only a short exposure to IVR was implemented (less than 6 min), to guarantee the participants´ safety, it would be interesting to increase the duration of the intervention, either in terms of the number of sessions per week, or the duration of each session, or both. With all these improvements, more significant benefits could be expected than those achieved in relation to the secondary objective of this pilot study. Therefore, improvements in the physical capacity of the participants could be achieved across a greater number of variants, and possibly more significantly, compared to those who do not carry out this type of work.

This pilot study has succeeded in verifying the feasibility and safety of IVR for people diagnosed with MS. However, for the reasons stated in the comments above, future research is required in pwMS in which longer interventions are carried out and in which there is the possibility of post-intervention follow-up, with larger sample sizes, and with IVR protocols of more sessions per week and/or longer sessions. As well as being useful and effective, the benefits of this exercise, together with a tool that allows the performance of therapeutic exercise sessions—indispensable for the selected population [7]—in an entertaining and fun way, result in patient motivation and adherence to treatment that is essential, and difficult to achieve with other tools or conventional methods.

## 5. Conclusions

The results obtained show that an 8-week IVR intervention is feasible and safe, as well as having beneficial effects for people with MS, and that it is a useful tool in motivating this population to take up physical exercise, as well as encouraging adherence to it. No adverse events were found during the sessions, and thus, they can be considered safe. Furthermore, users rated the IVR experience very positively and with excellent usability. Therefore, the implementation of a boxing or similarly themed IVR exergame could be considered as a complementary physiotherapy treatment to other techniques and as a valid and enjoyable way to carry out therapeutic exercise for people with MS.

In order to adequately study the possible physical, clinical and functional effects of IVR use in people with MS, future research is needed, with representative sample sizes that can be generalised for the rest of the population and with interventions of longer duration, both in terms of IVR exposure time, and the number of sessions per week and/or the number of weeks.

## Figures and Tables

**Table 1:** Sociodemographic characteristics and functional capacities of the sample at baseline.

		EG (*n* = 8)				CG (*n* = 10)				Independent *t*-Test
		Mean/%	SD	Min.	Max.	Mean/%	SD	Min.	Max.	
Age (years)		41.13	4.88	33	48	48.20	5.40	36	51	F = 9.339; Sig = 0.008
Sex (Female)		75	-	-	-	70	-	-	-	-
Diagnostic time (years)		8.75	7.42	22	11	14.80	8.83	28.00		F = 0.614; Sig = 0.071
Tinetti (points)	Balance	15.13	1.25	13.00	16.00	14.40	1.96	11.00	16.00	F = 3.557; Sig = 0.149
	Gait	11.00	1.41	9.00	12.00	10.80	1.14	9.00	12.00	F = 1.434; Sig = 0.249
	Total	26.13	2.10	23.00	28.00	25.20	2.94	21.00	28.00	F = 2.932; Sig = 0.106
FTSST (s)		14.42	4.51	10.38	23.68	12.84	3.79	7.54	19.32	F = 0.162; Sig = 0.693
TUG (s)	Simple	10.69	5.64	5.58	19.28	8.01	1.93	5.47	10.81	F = 10.969; Sig = 0.004
	Cognitive	12.00	5.79	5.84	21.37	9.30	2.63	5.71	13.09	F = 3.366; Sig = 0.085
FSS (points)		38.50	13.43	18.00	57.00	41.20	12.57	21.00	57.00	F = 0.038; Sig = 0.848
Handgrip (kg)		42.07	9.06	32.70	61.90	43.07	4.29	34.80	50.30	F = 1.292; Sig = 0.272
Reaction time (ms)		565.88	78.35	470.00	718.00	576.40	149.36	386.00	880.00	F = 2.811; Sig = 0.113

CG: Control Group; EG: Experimental Group; FSS: Fatigue Severity Scale; FTSST: Five Times Sit-to-Stand Test; kg: Kilograms; Max: Maximum; Min: Minimum; ms: Milliseconds; *n*: Sample; s: Seconds; SD: Standard Deviation; TUG: Timed Up and Go Test.

**Table 2:** Final results on usability, safety and post-game experiences.

	Experimental Group		
	Mean	Minimum	Maximum
SUS	90.31/100	72.5	100
SSQ	1.37/48	0	6
GEQ (positive experiences)	3.10/4	2	4
GEQ (negative experiences)	0/4	0	0
GEQ (fatigue)	0.43/4	0	1.5
GEQ (return to reality)	0.16/4	0	1

GEQ: Game Experience Questionnaire; SSQ: Simulator Sickness Questionnaire; SUS: System Usability Scale.

**Table 3:** Results of exergame, perceived exertion and Cybersickness in sessions 1 and 16.

	Grupo Experimental	
	Session 1	Session 16
Score (mean)	103,385.37	148,548.12
% correct score (mean)	40.72	58.48
Borg (mean)	3.62	2.37
Cybersickness (mean)	0.25	0

**Table 4:** Outcomes in functional capacities (pre-post intervention values in both groups).

			EG (*n* = 8)		CG (*n* = 10)
		Pre Mean ± SD	Post Mean ± SD	Pre Mean ± SD	Post Mean ± SD
Tinetti (points)	Balance	15.13 ± 1.25	15.25 ± 0.89	14.40 ± 1.96	14.40 ± 1.26
	Gait	11.00 ± 1.41	11.38 ± 0.92	10.80 ± 1.14	11.00 ± 1.15
	Total	26.13 ± 2.10	26.63 ± 1.51	25.20 ± 2.94	25.40 ± 2.27
FTSST (s)		14.42 ± 4.51	12.74 ± 4.67	12.84 ± 3.79	12.07 ± 2.79
TUG (s)	Simple	10.69 ± 5.64	9.24 ± 4.69	8.01 ± 1.93	8.66 ± 2.26
	Cognitive	12.00 ± 5.79	9.69 ± 5.07	9.30 ± 2.63	10.23 ± 3.17
FSS (points)		38.50 ± 13.43	36.00 ± 14.99	41.20 ± 12.57	39.70 ± 10.88
Handgrip (kg)		42.07 ± 9.06	48.51 ± 17.20	43.07 ± 4.29	51.25 ± 5.83
Reaction time (ms)		565.88 ± 78.35	472.38 ± 134.32	576.40 ± 149.36	527.90 ± 127.23
Borg (points)		2.75 ± 2.25	2.88 ± 0.99	-	-

CG: Control Group; EG: Experimental Group; FSS: Fatigue Severity Scale; FTSST: Five Times Sit-to-Stand Test; kg: Kilograms; ms: Milliseconds; *n*: Sample; s: Seconds; SD: Standard Deviation; TUG: Timed Up and Go Test.

**Table 5:** Pre- and post-intervention differences between groups in functional capacities (paired *t*-test, ANOVA 2 × 2).

		Intragroup (Pre-Post)		Intergroup ANOVA 2 × 2 (Moment × Group)		
		EG (*n* = 8) Sig.	CG (*n* = 10) Sig.	Group Sig.	Measurements Sig.	Interaction Sig.
Tinetti (points)	Balance	0.685	1.000	0.467	0.467	0.824
	Gait	0.800	0.443	0.110	0.897	0.897
	Total	0.170	0.662	0.175	0.655	0.848
FTSST (s)		0.042 *	0.355	0.040 *	0.361	0.731
TUG (s)	Simple	0.009 *	0.072	0.018 *	0.518	0.455
	Cognitive	0.003 *	0.181	0.036 *	0.370	0.284
FSS (points)		0.192	0.618	0.465	0.647	0.909
Handgrip (kg)		0.108	0.001 *	0.577	0.035 *	0.794
Reaction time (ms)		0.092	0.225	0.444	0.105	0.601
Borg (points)		0.885	-	-	-	-

CG: Control Group; EG: Experimental Group; FSS: Fatigue Severity Scale; FTSST: Five Times Sit-to-Stand Test; kg: Kilograms; Max: ms: Milliseconds; *n*: Sample; s: Seconds; SD: Standard Deviation; Sig: Significance; TUG: Timed Up and Go Test; * *p* > 0.05.

## Data Availability

The raw data supporting the conclusions of this article will be made available by the authors upon request.
